# Applications of Wild Isolates of *Saccharomyces* Yeast for Industrial Fermentation: The Gut of Social Insects as Niche for Yeast Hybrids’ Production

**DOI:** 10.3389/fmicb.2020.578425

**Published:** 2020-10-29

**Authors:** Monica Di Paola, Niccolò Meriggi, Duccio Cavalieri

**Affiliations:** Department of Biology, University of Florence, Florence, Italy

**Keywords:** *Saccharomyces cerevisiae*, social insects, yeast–insect association, hybrids, wine, beer, biotechnological application, ecology

## Abstract

In the industry of fermented food and beverages, yeast cultures are often selected and standardized in order to ensure a better control of fermentation and a more stable product over time. Several studies have shown that the organoleptic characteristics of fermented products reflect geographic variations of the microbial community composition. Despite investigations of the worldwide distribution and genetic diversity of *Saccharomyces cerevisiae*, it is still unclear how and to what extent human intervention has shaped the brewer’s yeast population structure. The genotypic and phenotypic characterization of environmental yeast populations and their potential application in the fermentative processes can significantly enrich the industrial fermentation products. Social insects have proven to be closely associated to the yeasts ecology. The relationships between yeasts and insects represent a fundamental aspect for understanding the ecological and evolutionary forces shaping their adaptation to different niches. Studies on phylogenetic relationships of *S. cerevisiae* populations showed genetic differences among strains isolated from gut and non-gut environments (i.e., natural sources and fermentation). Recent evidences showed that insect’s gut is a reservoir and an evolutionary niche for *Saccharomyces*, contributing to its survival and evolution, favoring its dispersion, mating and improving the inter-specific hybrids production during hibernation. Here, we discuss the potential use of social insects for production of a wide range of hybrid yeasts from environmental *Saccharomyces* isolates suitable for industrial and biotechnological applications.

## History of Fermentation and Yeast Domestication

**FIGURE 1:**
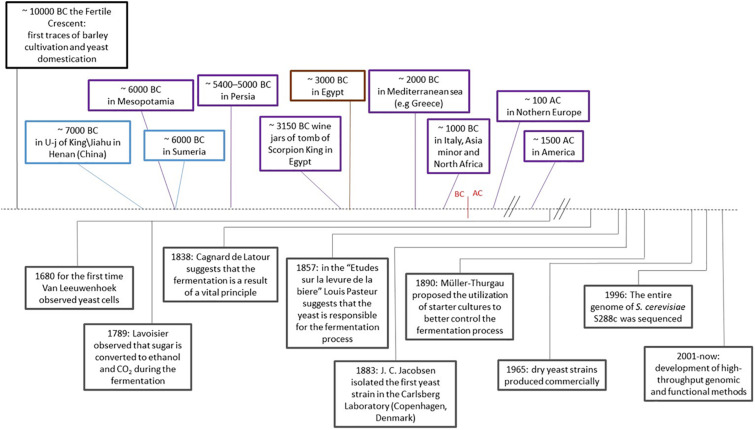
History of fermentation and yeast domestication. *S. cerevisiae* and human activities related to the production of fermented products. In the timeline: on the top of the dashed line, the oldest traces of *S. cerevisiae* across the human history and geography. Blue: fermented products; purple: wine; brown: bakery products. Below the dashed line, events related to *S. cerevisiae* in the history of fermentation and scientific advancements.

Many species of Saccharomycetes and non-Saccharomycetes are essential components of human production of fermented food and beverages. *Saccharomyces cerevisiae*, known as the brewer’s or bakery’s yeast, has been widely used for its fermentative capacity for thousands of years (Legras et al., 2007) (Figure 1). The fermentation process has allowed preservation of perishable food and made bioavailable nutrients and microelements, improving the quality of foods (Hatoum et al., 2012). The oldest traces of yeast and fermented foods have been found in the tomb of U-j of King/Jiahu in Henan in China (7,000 B.C.) (McGovern et al., 2004), in Iran (6,000 B.C.) (Fatahi et al., 2003), in wine jars of tomb of Scorpion King in Egypt (3,150 B.C.) (Cavalieri et al., 2003), and in Mesopotamia (about 4,000 to 3,100 B.C.) (McGovern, 2003), demonstrating that fermentation of cereals, honey and fruit, has been carried out since the dawn of civilization (McGovern et al., 2004; Kupfer, 2013, 2015). Successively, viticulture spread in Asia Minor and northern Africa, and around 1,000 B.C. arrived in Mediterranean countries (Legras et al., 2007). Beer production is supposed to be almost as ancient as wine and came from the Middle East, subsequently acquired by Germanic and Celtic populations around 1st century A.C. (Legras et al., 2007).

The long lasting association of *S. cerevisiae* with fermentative processes has led to propose the idea that its wide use caused its domestication as adaptation to different fermented products (Legras et al., 2007; Sicard and Legras, 2011). Yet the effect of domestication on trait selection has been proven only in the yeast strains used for the beer production (Piskur et al., 2006; Berlowska et al., 2015; Gallone et al., 2016, 2018).

The art of fermentation had been developed empirically from generation to generation. Scientific awareness related to biochemical transformations during fermentation started around the end of the 18th and in 19th century (Gay-Lussac, 1815; Kützing, 1837; Cagniard-Latour, 1838). In 1883, the first pure yeast culture was created by Emil Christian Hansen for beer production (Figure 1). Hansen had been brought to work from Copenhagen University to the Carlsberg’s Laboratories by Jacobsen, the founder of the Carlsberg Empire, to standardize the quality of beer by isolating and stabilizing the microorganism used in the brewing process. Subsequently in 1890, Hermann Mueller-Thurgau planned the process for a better control and repeatability of wine fermentations with starter cultures (Marsit and Dequin, 2015) (Figure 1). The rise of the industry of fermented products and the application of innovative biotechnological methods for selection of enhanced yeast strains or production of hybrids suitable for different types of fermentable substrates enables a much more defined control of the fermentation process than the generally used strains.

## Social Insects and Yeasts of Natural Environments

The ability of yeasts to metabolize sugars by producing ethanol through anaerobic fermentation even when oxygen is available (the Crabtree effect) (Chambers and Pretorius, 2010) and in presence of high glucose concentrations (Otterstedt et al., 2004) has enabled *S. cerevisiae* to gain an evolutionary advantage over other microorganisms (Albergaria and Arneborg, 2016). Ethanol production and acidification of the growth medium have always kept bacteria and other aerobic molds under control. The microbial communities, including yeasts, can play a key role in triggering fermentation processes conferring a typical bouquet, thanks to volatile compounds (El-Sayed et al., 2005; Christiaens et al., 2014). *S. cerevisiae* produces several aromatic esters: ethyl acetate (varnish, nail polish, and fruity aroma), isoamyl acetate (banana and pear), isobutyl acetate (banana), phenylethyl acetate (fruity and flowery), ethyl hexanoate (apple, banana, and violets), ethyl octanoate (pineapple and pear), and ethyl decanoate (floral) (Ruiz et al., 2019).

The question of the natural environmental niche for *S. cerevisiae* was debated for a long time (Goddard and Greig, 2015). Environmental *S. cerevisiae* strains are subjected to harsh conditions and they developed survival strategies, which are not retained when laboratory or industrial strains are cultured under most favorable conditions. *S. cerevisiae* has been isolated from different natural environments, such as oak trees (Sampaio and Goncalves, 2008; Zhang et al., 2010; Hyma and Fay, 2013), maize (Hayford and Jespersen, 1999; Halm et al., 2004), various fermentations and other substrates, including soil (van der Aa Kuhle et al., 2001; Oba et al., 2011; Dunn et al., 2012; Kubo et al., 2014).

*Saccharomyces cerevisiae* has adapted itself and evolved to different environmental niches (Goddard and Greig, 2015; Peter et al., 2018). Evidences indicated that, although *S. cerevisiae* is found in abundance in environments, such as wineries, it does not originate from grapevines or grape berries. Mortimer and Polsinelli (Mortimer and Polsinelli, 1999) demonstrated that *S. cerevisiae* was found at very low frequency on unripe and intact grape berries (0.05% on average), while during grape maturation it was present on average with a frequency of 25% on broken berries (Mortimer et al., 1994; Polsinelli et al., 1996; Mortimer and Polsinelli, 1999). Thus, ripe and crushed grape berries (with high concentration of fermentable sugars), represent a suitable environment for *S. cerevisiae*. In a recent study (Taylor et al., 2014) the rare presence of *S. cerevisiae* in intact grapes was confirmed: one *S. cerevisiae* cell was found among 20,000 cells of other fungi by metagenomic approach.

Researchers wondered how *S. cerevisiae* could be carried on the grapes. The agents proposed to play a role in spreading of microorganisms in the environment are animal vectors (e.g., insects and birds) (Mortimer and Polsinelli, 1999; Goddard et al., 2010; Stefanini et al., 2012). Unlike bacteria and fungal spores that can easily be dispersed by other means (e.g., air/wind) (Madden et al., 2018), yeast spores are not adapted for wind-borne transmission. Many decades ago (Grace and Collins, 1976), testing the dispersion rate of bacteria and yeasts spores using a wind tunnel, demonstrated that the wind is unable to disperse *S. cerevisiae* cells when these are adhered to the leaf surface.

Evidences showed that insects have a mutualistic relationship with yeasts (Belisle et al., 2012; Stefanini et al., 2012, 2016; Madden et al., 2018) and play a key role for yeast dispersion in natural environments. *Saccharomyces* spp., in particular *S. cerevisiae*, were detected in different insects worldwide (Stefanini et al., 2012; Buser et al., 2014; Jimenez et al., 2017; Meriggi et al., 2020; YeastFinder^1^; Table 1).

**TABLE 1:** Isolation of *Saccharomyces* spp. in the insects’ gut and geographical distribution.

**Insect order**	**Insect common name**	**Yeast species**	**Geographical distribution**	**References**
Hymenoptera	Wasps Honeybees	*Saccharomyces cerevisiae Saccharomyces ludwigii*	North America, Brasil, Europe, and New Zeland	Batra et al. (1973), Sandhu and Waraich (1985), Stefanini et al. (2012), Jimenez et al. (2017), and Meriggi et al. (2019)
Diptera	Fruit flies Flies	*Saccharomyces cerevisiae Saccharomyces ludwigii*	New Zeland, Australia, Taiwan, Seychelles Islands, Brasil, and Europe	Phaff and Knapp (1956), Kircher et al. (1982), Broderick and Lemaitre (2012), Buser et al. (2014), and Meriggi et al. (2019)
Coleoptera	Beeteals	*Saccharomyces cerevisiae*	North America, Asia, and Africa	Suh et al. (2005) and Stefanini (2018)

Social insects represent a fulcrum in the yeast ecology and evolution. At the same time, volatile compounds produced by yeasts attract insects that preferentially foraged nectar sources, and influence their behavior and physiology (Becher et al., 2012; Babcock et al., 2017; Stefanini, 2018).

In our previous studies (Stefanini et al., 2012, 2016), we demonstrated not only that wasps contribute to dispersion of yeast strains into the environment, but also that they can host yeasts in their gut, contributing to their survival and biodiversity. *Polistes dominula* allows the transmission of yeasts to the progeny (Stefanini et al., 2012), ensuring the presence of yeast in the colony and in the foraging area. This make a flow of yeast cells in the environments (in the vineyard, on grapes) from autumn to spring. These observations allow to explain where yeasts can reside during the winter and how reappear during the spring and summer, an unsolved question up until few years ago.

In spite these evidences, specific factors that select natural yeasts in the insect gut have not yet been found. Our recent findings showed that yeast has evolved strategies to adapt to the gastrointestinal tract of insects (Stefanini et al., 2012, 2016; Ramazzotti et al., 2018). The wall of the ascospore is able to resist to the insects gastric digestion, allowing survival within the host gut (Coluccio et al., 2008; Stefanini et al., 2012, 2016). More recently, we demonstrated that host’s gut, not only of insect but also human, is a potential reservoir for yeasts (Ramazzotti et al., 2018). Genetic and phenotypic differences, including peculiar cell wall composition, different ability to sporulation and to induce host immune response were discovered between strains isolated from human and insect’s gut and non-gut environment, suggesting the existence of gut-specific features that could represent a selective advantage for survival and expansion in the gut environment (Ramazzotti et al., 2018).

Based on our previous studies (Stefanini et al., 2012, 2016), the insect’s gut is an advantageous ecological niche for *S. cerevisiae*, favoring the intra− and inter−species mating of yeast cells, and allowing increased fitness of hybrids, thus representing an environment favoring the generation of yeast genetic biodiversity.

The current used industrial strains represent a small fraction of the natural biodiversity (Liti et al., 2009). The nature could provide unknown strains with relevant characteristics that may enhance the industrial fermentations. Specific strategies could help to transfer these properties to industrial strains or create novel strains with best performance for fermentation processes. Here, we propose and discuss the use of wasp’s gut for accelerated selection and production of hybrids suitable for different types of fermentable substrates.

## Yeast Strains and Fermentation Industry: Hybrids Do It Better

Recent genomic studies provided a comprehensive overview of the biodiversity of wild and industrial *Saccharomyces* strains (Liti et al., 2009; Schacherer et al., 2009). Diversity within *S. cerevisiae* population structure was at least in part associated to its industrial application. According to the domestication hypothesis, this long-term process has resulted in different strains with specific characteristics suited for industrial fermentation, clustering differently from wild populations (Fay and Benavides, 2005; Liti et al., 2009; Schacherer et al., 2009; Sicard and Legras, 2011). Several studies reported genome-wide signatures of clonal expansion of yeast strains, as well as convergent evolution of industrially relevant traits in separate lineages (Liti et al., 2009; Gallone et al., 2016; Parapouli et al., 2020). Yet the only *bona fide* evidence for selection on genomic regions associated to domestication was reported by Gallone et al. (2019) that studied the whole-genome of more than 200 industrial yeasts showing that about 25% consisted of interspecific hybrids derived from *S. cerevisiae*, *S. kudriavzevii*, *S. eubayanus*, and *S. uvarum*. Langdon et al. (2019) analyzed the genomes of 122 interspecies hybrids and introgressed strains in *Saccharomyces* genus revealing three domesticated lineages, including wild lineages from Europe and Northern continents of the world. These evidences show that industrial yeasts are the result of selection following clonal expansion and adaptation of specific strains, shaped by genetic drift caused by bottlenecking.

Beer’s yeasts present the strongest and maybe only genetic and phenotypic signatures of domestication (Gallone et al., 2019; Langdon et al., 2019). The strong selective pressure imposed over many generations allowed to obtain desirable phenotypes, but has also dramatically affected the genomic structure and stability of domesticated yeasts (Legras et al., 2007; Gallone et al., 2016, 2018). Hybrid strains are preferred in the industrial fermentation because they show phenomena, such as “vigor of the hybrid” that confer better fermentation capacity in terms of speed, use of alternative sugar source and an enriched pattern of aromatic compounds (Bellon et al., 2011; Piotrowski et al., 2012; Bellon et al., 2013; Gamero et al., 2013; Snoek et al., 2015).

An example of yeast’s domestication trait is the ability to ferment maltotriose. This trait evolved independently and through different genetic pathways in the two main beer lineages, such as ale (by the top-fermenting *S. cerevisiae*) and lager (by the bottom – fermenter *S. pastorianus*, a interspecific hybrid *S. cerevisiae* × *S. eubayanus*), suggesting strong selection pressure (Gallone et al., 2016, 2018). *S. pastorianus* can ferment at lower temperatures than *S. cerevisiae* (Dunn and Sherlock, 2008; Libkind et al., 2011). This hybridization process has combined the efficiency of *S. cerevisiae* in the sugar metabolism and the cryoprotective capacities of *S. eubayanus* (Hebly et al., 2015; Krogerus et al., 2015). The cryoprotective capacity was demonstrated to be related with the mitochondrial genome inheritance. *S. eubayanus* (Baker et al., 2019) and *S. uvarum* (Li et al., 2019) mitochondrial genome provided with low-temperature tolerance to the interspecies hybrids compared when the same hybrids inherited the mitochondrial genome of *S. cerevisiae*. In addition, hybrids between natural strains of *S. kudriavzevii* × *S. cerevisiae*, selected to confer diverse flavors, are used for the production of Trappist beers (a subgroup of ale) (Gonzalez et al., 2008; Peris et al., 2018; Gallone et al., 2019; Langdon et al., 2019).

On the other hand, several authors suggested that during centuries of wine production, *S. cerevisiae* acquired remarkable resistance/tolerance to high sugar concentrations and nitrogen metabolic activity, through adaptive horizontal gene transfer and copy number variations (Fay and Benavides, 2005; Aa et al., 2006; Ezov et al., 2006; Ruderfer et al., 2006; Legras et al., 2007; Stefanini and Cavalieri, 2018). These events potentially conferred competitive advantages during must fermentation (Almeida et al., 2017), and production of a wide spectrum of aromatic profiles (González et al., 2007). Triple hybrid *S. cerevisiae* × *S. kudriavzevii* × *S. uvarum* has shown to be able to use fructose more efficiently than *S. uvarum* strain and to restart fermentation (Christ et al., 2015), avoiding the upper hand of bacteria and the consequent spoilage of the must. Inactive or stuck fermentations are detrimental to wine/beer production. These events have been reduced by the commercial availability of selected yeast strains used as starter. However, this practice limits the developing of wild yeasts during fermentation (Parapouli et al., 2020). Camarasa et al. (2011) investigated the fermentative efficiency of a set of *S. cerevisiae* strains, observing that strains isolated from sugar-rich environments were able to complete the fermentation process, while the laboratory or environmental strains were unable. The extensive genetic diversity of environmental *S. cerevisiae* isolates, in particular of interspecific hybrids, could be an extremely significant source of innovation for biotechnological applications (Parapouli et al., 2020).

## Biotechnological Applications of Insects as Yeast Hybrids’ Producers

The quest for increased and improved productivity and adaptability to changing consumer preferences lead to the study and development of industrial strains with novel and desired properties.

Non-genetically modified organisms (non-GMO) and GMO techniques can be used for selection of yeast strains with suitable traits and industrially relevant phenotypes. Jan Steensels et al. (2014) extensively described these approaches, indicating their advantages and limitations. Non-GMO techniques have been developed to create performant yeast variants, that can be freely used in industrial fermentations, without encounter any problems with legislation and/or consumer acceptance.

Currently there are four main approaches for generate artificial diversity in yeast strain using sexual hybridization: (i) direct mating – crossing of two haploid cells or spores of opposite mating types; (ii) rare mating – crossing of strains without sporulation by occasional and rare homothallic mating-type switch; (iii) mass mating – crossing of multiple parental strains or a heterogeneous population of the same parental strain; and (iv) genome shuffling of multiple strains. In addition, asexual hybridization includes cytoduction (a method that transfer cytoplasmically inherited traits) and protoplast fusion (asexually merging of cells after cell wall distruption in osmotic medium). Overall, the above mentioned approaches could have important impact in the optimization of industrial processes, at technical and economic level. Our previous studies demonstrated that the insects’ gut represents the environment where *S. cerevisiae* mates and interspecific hybrids arise spontaneously. After 2 and 4 months of hibernation in the wasp’s gut, *S. paradoxus* can survive only in hybrid shape with *S. cerevisiae*. The rate of inbreeding in *S. cerevisiae* spores increases up to ten times when inoculated in the insect gut. The high frequency of outbreeding coincides with the rates of mosaicism and genetic diversity in yeast strains (Reuter et al., 2007).

In last years, the interest for insects for food and applicative purposes is growing. The insects breeding is a sustainable method with low energy impact and represents a great opportunity for large-scale industrial applications. Social insects could play a role in the evolution and genetic recombination of yeasts. They seems to be a perfect niche in which the formation of hybrids occurs naturally and much more efficiently than in any other wild place, without employing artificial strategies of genetic manipulation, as recently showed by Peris et al. (2020) making synthetic hybrids by six yeast species. Wasps could provide yeast communities with the level of genetic variation required in time to adapt to a changing environment. Given the fast pace at which climate change is affecting the man made, “ersatz” environment where fermented products are produced, maintaining the genetic variation force driven by the presence of insects could be very important to support the adaptability of yeast to these changes. Considering that many yeasts used in the fermentation industry are interspecific hybrid strains, we could speculate that insects have been the breeders of the past, having likely provided a great deal to the evolution of brewing and wine making. The need of product diversification present in the fermented beverages field could look with great interest at the potential of using wasps as breeding places to produce yeasts of biotechnological interest, including yeasts producing beverages with a reduced ethanol content.

## Conclusion

Optimization of current strategies and novel technologies such as *next-generation sequencing*, together with a better understanding of complex phenotypes allows to create yeasts with more variants and better adapted to the industrial goals. In the near future, the fermented beverages industry could benefit significantly from the possibility to breed insects massively and use them as a forge for accelerated selection and production of hybrids suitable for different types of fermentable substrates, further modeling these according to biotechnological requirements.

We can conclude that the yeasts–insect association certainly goes beyond the simple link between vectors and transported. In a future perspective, a better understanding of the ecology and relationships between insects and yeasts can play a key role in producing fermented beverages meeting the needs of tomorrow’s consumers.

## Author Contributions

MDP, DC, and NM ideated the review. MDP and NM gathered the data. MDP, NM, and DC wrote the manuscript. All authors contributed to the article and approved the submitted version.

## Conflict of Interest

The authors declare that the research was conducted in the absence of any commercial or financial relationships that could be construed as a potential conflict of interest.

## Footnotes

1 www.stefaninilab.com/tools/
